# 

**Published:** 1817-04

**Authors:** 


### CATALOGUE OF MEDICAL BOOKS.

Observations on the Gout and Acute Rheumatism. By C. Wilson, M.D. &c. 2d edition; small 8vo.

A Descriptive Catalogue of recent Shells, arranged according to the Linnæan Method. By Lewis Weston Dillern, F.R.S. &c. 2 vols. 8vo.

Outlines of Geology, the Substance of a Course of Lectures delivered in the Theatre of the Royal Institution. By William Thomas Brande. 8vo.

Cursory Remarks on a Bill now in the House of Peers "For Regulating of Mad-houses;" its probable Influence upon the Physical and Moral Condition of the Insane, and upon the Interests of those concerned in their Care and Management. By George Man Burrows, M.D. F.L.S. &c.

### FOREIGN BOOKS LATELY PUBLISHED.

De Duplicitate Monstrosa Commentarius, quem Conscripsit Joannes Fredericus Meckel, Med. Utriusq. Dr. Anatomial Zoologiæ et Physiologiæ, Prof. P. O. Societat. Complur. Doctur. Socius. Ordinis Wladimiriani Eques. Acad. Tabul. acnea VIII.

The Disorders of the Heart, systematically arranged and elucidated by Observation. By Dr. F. L. Kreysig, Physician and Aulic Counsellor to his Majesty the King of Saxony. The Second Part of the Second Volume.

Elements of Pharmaceutical Chemistry, for the Use of Physicians and Apothecaries. By Dr. J. W. Döbereiner, Prof. of Chemistry, Pharmacy, and Technology, in the University of Jena, &c.

On the Advantages to be derived from the Russian Vapour Baths and Sweating Baths, and on their Construction. By Dr. Ch. Fred. Hirsch, Royal Bavarian Medicinal Counsellor, &c.

Chemical Analysis of the Sulphureous Well of Gun Sherbad, near Sondershausen, with its Description in Topographical, Economical, and Medical Respect; with an Appendix, containing the Chemical Analysis of the Muriatic Wells at Stockhawen. By Chr. Fr. Bucholy, Doctor of Pharmacy and Philosophy, and Prof. at Erfurt, &c.

---

### *NOTICES TO CORRESPONDENTS.*

*In conformity with* Mr. Ward's *earnest wishes, from the best of motives, we have inserted his paper this month; but, in order to find room, have been obliged to shorten it, trusting to his candour for our excuse.*

*Several favours from Correspondents will appear in our next.*

